# Creating a Comprehensive Pandemic Response to Decrease Hospitalist Burnout During COVID-19: Intervention vs Control Results in 2 Comparable Hospitals (HOSP-CPR)

**DOI:** 10.1007/s11606-023-08041-6

**Published:** 2023-02-10

**Authors:** Tricia T James, Robert Hudon, Todd Merrick, Lisa Olson, Douglas Hanes, James M. Scanlan

**Affiliations:** 1 Department of Medical Education, Providence Portland Medical Center, 5050 NE Hoyt Suite 540, Portland, OR 97213 USA; 2 Department of Hospital Medicine, Providence Portland Medical Center, Portland, OR USA; 3 Center for Cardiovascular Analytics, Research + Data Science, Providence Research Network, Portland, OR USA; 4 Providence Health Research Accelerator (HRA), Seattle, WA USA

**Keywords:** burnout, COVID-19, wellness, wellness leaders, weekly meetings, coaching, support group

## Abstract

**Background:**

Physician burnout increased during the COVID-19 pandemic.

**Objective:**

To evaluate the effectiveness of a multimodal workplace intervention designed to reduce hospitalist burnout.

**Design:**

*Participants and setting*: Our intervention group was composed of internal medicine hospitalists at Providence Portland Medical Center (64 providers including 58 physicians and 6 nurse practitioners). Our control was composed of internal medicine hospitalists at Providence St Vincent’s Hospital (59 physicians and 6 nurse practitioners). *Measurements*: Two surveys were given during, before, and after a 12-month intervention period (October 2020 and again in October 2021). Surveys included demographics, job satisfaction, the Maslach Burnout Inventory, the Pandemic Experiences Survey, and 2 questions about leaving the job. *Interventions*: Three hospitalists designated as wellness warriors created weekly COVID group meetings, providing up-to-date information about COVID-19 infection rates, treatments, and work-flow changes. Discussions included coping and vaccine hesitancy, difficult case debriefs, and intensive care unit updates. Individual coaching was also offered. Meeting minutes were taken and sessions were recorded for asynchronous access.

**Results:**

No site differences in burnout or job satisfaction were evident pre-intervention. Post-intervention, the intervention group reported 32% burnout while controls reported 56% (*p* = .024). Forty-eight percent of the intervention group reported high wellness support vs. 0% of the controls (< .001). Intervention participants attributed 44% of wellness support to Providence alone, vs. controls at 12% (< .001). Regressions controlling sex, work hours, experience, race, and children in the home showed the intervention’s positive effects on burnout and job satisfaction remained significant (all *p* < .02).

**Limitations:**

For privacy reasons, all survey responses were anonymous, meaning that individual pre-post changes could not be tracked.

**Conclusion:**

We believe the intervention resulted in substantial burnout prevention and is feasible for adoption in most hospitals and clinics.

## Background

Burnout is an occupational distress syndrome which includes emotional exhaustion, depersonalization, and a decreased sense of personal accomplishment.^1^ Burnout is associated with increased medical errors, patient mortality, depression, suicidal ideation, and job turnover.^2–5,7^ Awareness of physician suffering had been increasing prior to the COVID-19 pandemic.^8^ US hospitalist well-being has decreased significantly compared to pre-pandemic levels.^9^ Because of hospital overloading due to the COVID pandemic, physician burnout is even more widespread than that pre-pandemic and risks becoming chronic. Physician burnout has recently been acknowledged as a public health crisis by the Surgeon General.^10^

Interventions that can reduce physician burnout are urgently needed. Research suggests that organizational change is more effective than individually targeted support in reducing medical burnout.^11,12^ However, burnout is a complex phenomenon with unique drivers in different groups, which makes developing effective interventions a formidable challenge. Most organizations lack both the structure to develop interventions and to assess their benefits. This has led to widespread perceived lack of agency for individuals and groups. Our goal was to evaluate the effectiveness of a multimodal intervention designed to reduce burnout in hospitalists during the COVID-19 pandemic. We hoped that it could also serve as a blueprint for other sites and situations.

## Methods

This study was approved by the Providence St. Joseph Health IRB.

### Subjects

One internal medicine hospitalist group (64 providers—58 physicians and 6 nurse practitioners) at Providence Portland Medical Center, a 483-bed hospital in Portland, OR, served as our intervention group. The internal medicine hospitalist group (65 providers—59 physicians and 6 nurse practitioners) at Providence St Vincent’s Hospital, a 523-bed hospital within the same system and city served as our control comparison. Both groups received and completed baseline surveys via email, and participation was completely voluntary. None of the control providers received experimental interventions.

### Interventions

The total project duration was 15 months. The initial 3 months (July 2020–September 2020) was devoted to preparation work that included identifying 3 hospitalist “wellness warriors,” based on expressed interest and willingness to commit the time needed for the study. They were each paid for 2 h/week to develop, coordinate, and implement interventions. This approach was based on the model of physician-organization collaboration as developed by the Mayo Clinic to empower individual work units in the *listen, act, develop* process.^13^ The team performed a literature review regarding best practices in burnout prevention and developed questions to be included in a baseline survey to identify the largest group challenges. The baseline survey question specified our goal and posed questions about work: “The primary goal of this project is to improve the hospitalist experience by identifying specific interventions. Below are seven domains that can contribute to work satisfaction or burnout. Please provide specific details or comments for each. Your individual responses will guide our interventions. We need your help, your input is critical.” The seven domains were as follows: workload and job demands; control and flexibility; efficiency and resources; organization culture and values; social support and community at work; work–life integration; and meaning in work. The team then reviewed these comments and categorized them into regional/national challenges, system challenges presenting advocacy opportunities, group-specific challenges, and inherent job stressors. We focused on the group challenges, which were in the locus of control, and on shared system challenges identified with leadership, to empower advocacy. The intervention was delivered, at one site, over the next 12 months (October 2020–September 2021). The primary investigator was paid for 8 h/week to educate the wellness warriors about current wellness approaches, and to help identify stressors and develop interventions. The primary investigator also became certified as a coach and offered one–one-one coaching to interested hospitalists. Online COVID groups were created as one of the primary interventions (see Appendix).

Other interventions included hosting dinners with the wellness warriors and the 3 hospitalist group leaders to get their input about group needs as well as check in on their own well-being. We had 4 dinners during the study period. We also provided 15-min massages, small tokens of acknowledgement, and lunch at 3 points during COVID surges to acknowledge the added stress. Community was fostered through social functions. We hosted two outdoor meet-ups for hospitalists and their families at a nearby park. For recent hires (within the last 2 ½ years—43%), we hosted a Zoom social happy hour recognizing their unique experiences and potential isolation. The larger system implemented Behavioral Health appointments as a wellness support during the intervention period, and these were available to both the intervention and control groups. (See Appendix.)

### Questionnaires

Participants at both sites completed a baseline survey in October of 2020 and a post-intervention survey in October of 2021. The survey consisted of demographic information including age, gender, ethnicity, relationship status, children < 18 years old at home, FTE, and number of years since residency graduation. We also included a job satisfaction scale between 1 and 100, the Maslach Burnout Inventory^1^ (MBI—used under license with Mind Garden Inc.), the Pandemic Experiences Survey (PES) (see Appendix), and 2 turnover intent questions: *In the last 9 months, how often have you considered leaving your job*? *How often do you review other job opportunities*? Burnout was defined as high emotional exhaustion and/or depersonalization. On the baseline survey, we also asked the intervention site to report specific drivers within each of the 7 domains of satisfaction or burnout as outlined by Shanafelt and Noseworthy.^3^ On the post-intervention survey, both groups were asked if their wellness had been supported within the last 12 months, and if that support had come from within the organization or from outside of it. We also added an open-ended question about what support has been most helpful and what they would like to see in the future. Finally, the intervention participants were asked which interventions they had participated in. For both surveys, a $100 Amazon gift card was randomly awarded to one person from each group for completing the survey. Participants could enter the drawing via a link to a separate survey where they could provide their name. All survey responses remained anonymous.

### Statistical Analysis

Study group characteristics are presented as mean (SD) or count (%), depending on the type of measure. Groups were compared using chi-square (categorical variables), Wilcoxon (small-scale ordinal responses), or independent *t* tests (job satisfaction and Maslach scores). Groups were compared both at pre-intervention (to support similarity of sites) and post-intervention (for intervention effects). Due to anonymity of responses, pre- and post-responses cannot be matched for individual respondents. Since pre- and post-intervention survey cohorts were not identical, we focused on the impact of the intervention on the post-intervention cohorts.

We conducted linear and logistic regressions of post-intervention data, as appropriate to the specific outcome, to assess (1) associations of demographic/work factors to overall or EE burnout, job satisfaction, and burnout subscale scores pre-intervention; (2) associations of demographic/work variables as well as the intervention to the same outcomes post-intervention; and (3) associations of participation in different intervention components to burnout and job satisfaction (in the intervention group only). Model residual examination did not indicate any points with extreme influence on results. All analyses were conducted in R v.4.0.5.

#### Role of the Funding Source

The Providence Portland Medical Foundation funded the study costs with a grant titled “Supporting the PPMC Hospitalist Group During the COVID-19 Pandemic” for $160,506.

## Results

The intervention and control groups were very similar to each other. The demographic characteristics and employment characteristics of respondents did not differ between facilities prior to or post-intervention (see Table 1). The initial survey response rates were 78.1% (Providence Portland Medical Center (PPMC), *n* = 50/64; October 2020) and 78.4% (Providence St Vincent’s (PSV), *n* = 51/65; October 2020). The follow-up survey response rates were 72.5% (PPMC, *n* = 50/69; October 2021) and 61.4% (PSV, *n* = 43/70; October 2021). During the study period, the number of COVID-positive patients admitted to PPMC was 2200 with an 11% mortality rate compared to PSV with 2080 admissions with a 9% mortality rate.

**Table 1:** Demographic and Employment Variables: Pre- and Post-intervention

	Variables	Pre-intervention			Post-intervention		
		SVH	PPMC	*p* value	SVH	PPMC	*p* value
		Freq. (%)	Freq. (%)		Freq. (%)	Freq. (%)	
Age	Under 40	20 (39%)	20 (39%)	0.887	13 (30%)	22 (44%)	0.247
	40–49	26 (50%)	23 (44%)		25 (58%)	20 (40%)	
	50–70	6 (11.5%)	7 (14%)		5 (12%)	8 (16%)	
Sex	Female	25 (48%)	25 (48%)	1	19 (44%)	24 (48%)	0.394
	Male	25 (48%)	24 (46%)		22 (51%)	26 (52%)	
	Prefer not to say	2 (4%)	1 (2%)		2 (5%)	0 (0%)	
Ethnicity	White/Caucasian	26 (50%)	37 (71%)	0.07	26 (61%)	41 (82%)	0.171
	Asian/Pacific Islander	16 (31%)	8 (15%)		11 (26%)	6 (12%)	
	Black/African American	3 (6%)	0 (0%)		0 (0%)	0 (0%)	
	Other	1 (2%)	1 (2%)		2 (5%)	1 (2%)	
	Prefer not to answer	6 (12%)	4 (8%)		4 (9%)	2 (4%)	
Are you in a relationship?	Yes	50 (96%)	40 (77%)	0.013	40 (93%)	42 (84%)	0.202
Do you have a child < 18 years?	Yes	31 (60%)	26 (50%)	0.563	29 (67%)	30 (60%)	0.516
Years of experience	5 or less	14 (27%)	21 (40%)	0.059	13 (30%)	20 (40%)	0.28
	6–15	30 (58%)	17 (33%)		21 (49%)	16 (32%)	
	> 15	8 (15%)	12 (23%)		9 (21%)	14 (28%)	
Work hours	Full time	43 (83%)	34 (65%)	0.113	35 (81%)	36 (72%)	0.343
	Part time	9 (17%)	16 (31%)		8 (19%)	14 (28%)	

**Fig. 1:**
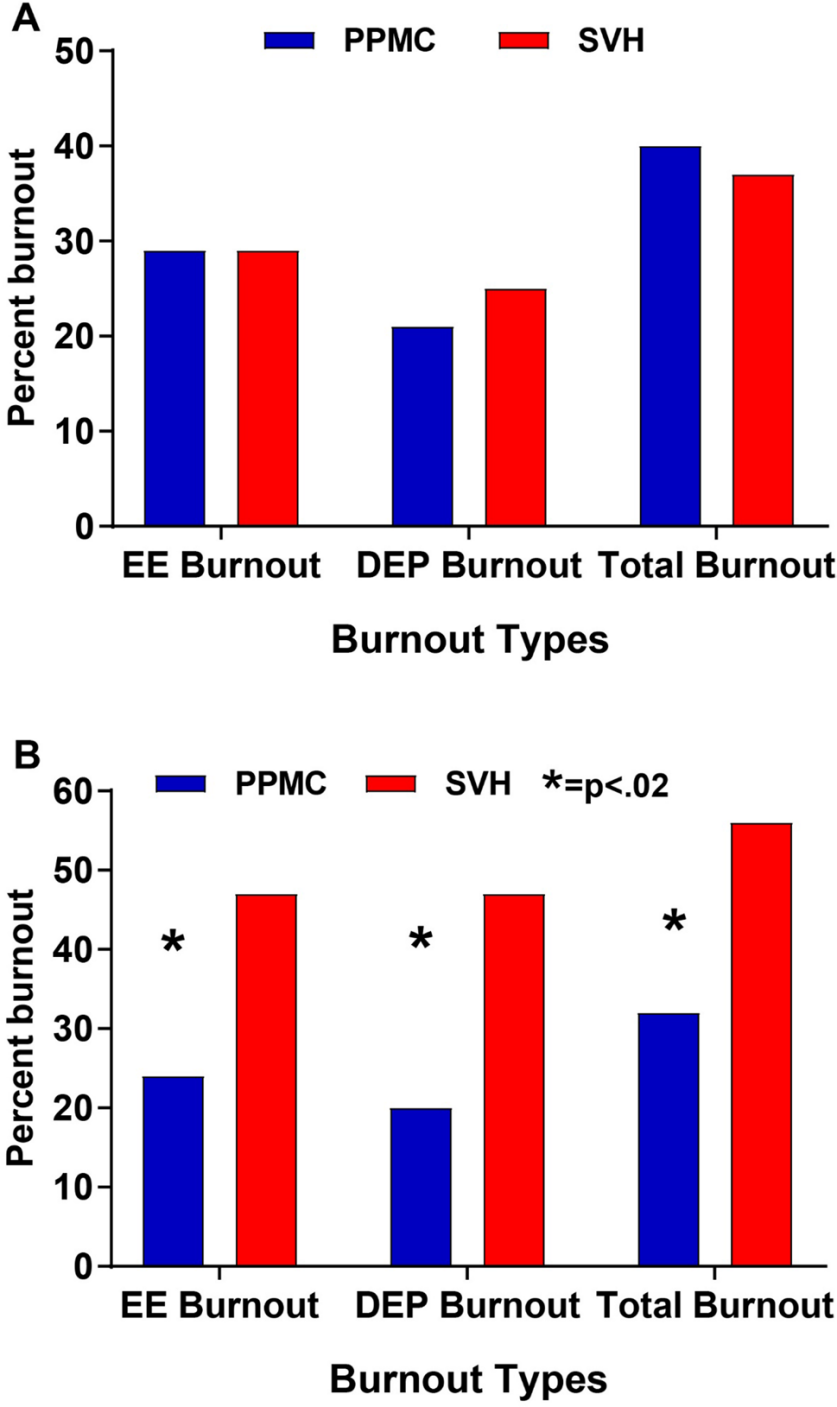
A Pre-intervention burnout by intervention (PPMC) and control (SVH) facilities. B Post-intervention burnout by intervention (PPMC) and control (SVH) facilities

We found no significant facility differences in any variables measured at the beginning of the study period, including all burnout measures (see Fig. 1A). However, after 1 year, the intervention group showed significantly lower emotional exhaustion, depersonalization, and overall burnout rates (see Fig. 1B and Table 2) and higher job satisfaction relative to controls. Personal accomplishment did not differ between groups. The Pandemic Experiences Survey revealed that the intervention group had increased feelings of appreciation, manageable work hours, organizational value alignment, and significantly higher perceptions of organizational leadership at follow-up (Table 2). The questions about leaving the job in the post-intervention survey did not differ significantly between the hospitals, but 5% of the control group reported “always” thinking about leaving vs 0% of the intervention group. In combined intervention and control samples, the intent to leave was most strongly related to job satisfaction (*r* = 0.47, *p* < 0.001), which in turn was strongly related to burnout (*r* =  − 0.61, *p* < 0.001).

**Table 2:** Post-intervention Assessment of Working Conditions, Caregiver Risk, and Occupational Burnout

	Variables	SVH	PPMC	*p* value
		*n* = 43	*n* = 50	
Has the pandemic affected (1 = no effect, 7 = large effect)	Your organization?	2 (0.7)	2 (0.7)	0.955
	Your work unit?	2 (0.6)	2 (0.7)	0.883
	You personally?	2.3 (0.9)	2.4 (0.9)	0.633
	Your work equipment?	1.5 (0.5)	1.3 (0.5)	**0.03**
Are the following adequate? (1 = completely adequate, 7 = completely inadequate)	Your equipment	1.5 (0.6)	1.5 (0.6)	0.858
	Support staff availability	2.6 (1)	2.3 (0.8)	0.249
	Support staff competence	2.1 (0.8)	2 (0.7)	0.829
	Information from management	2.2 (0.7)	1.9 (0.5)	**0.011**
Risk perception during pandemic (1 = no risk, 7 = life threatening)	To self	4.6 (1.2)	4.2 (1.1)	0.135
	To family	4.3 (1.3)	4.2 (1.4)	0.76
	To patients	5 (1.6)	4.7 (1.5)	0.248
	To colleagues	4.4 (1.5)	4.1 (1.2)	0.218
During the pandemic, did you	Have direct COVID19 contact? (1 = never, 5 = every day)	2.5 (0.9)	1.9 (1)	**0.006**
	Training and support – > COVID-19 control (1 = none, 5 = complete)	3.5 (0.6)	3.8 (0.5)	**0.012**
	Have danger from COVID19? (1 = life threating, 5 = no danger)	2.7 (0.9)	2.8 (0.9)	0.518
	Have manageable hours (1 = strongly disagree, 5 = strongly agree)	3.4 (0.9)	4 (0.8)	** < 0.001**
	Work in competence area	3.8 (0.8)	4 (0.8)	0.221
	Feel appreciated	3.4 (0.9)	3.9 (0.9)	**0.005**
	Feel social support	4.2 (0.7)	4.2 (0.9)	0.337
	Organization and personal values consistent	3.3 (0.9)	3.9 (0.9)	**0.001**
During the pandemic organizational leadership	Improved capabilities (1 = never, 5 = frequently)	3.6 (0.8)	4.2 (0.7)	** < 0.001**
	Expressed confidence in me (1 = never, 5 = frequently)	3.8 (0.9)	4.3 (0.6)	**0.005**
	Increased feeling of safety (1 = never, 5 = frequently)	3.5 (1.1)	4.2 (0.7)	** < 0.001**
	Were honest (1 = Never, 5 = Frequently)	3.5 (1.1)	4.2 (0.8)	** < 0.001**
	Work was meaningful (1 = strongly disagree, 5 = strongly agree)	4 (0.6)	4.2 (0.7)	0.267
	Treatment was satisfactory (1 = strongly disagree, 5 = strongly agree)	3.9 (0.6)	4.2 (0.8)	**0.034**
Has your wellness been supported in the last 12 months?	Yes (could not ask for anything more = 1, mostly/somewhat = 0)	0 (0%)	24 (48%)	< .001
Has Providence or outside people supported wellness?	Providence	5 (12%)	22 (44%)	< .001
	Both within and outside Providence	18 (42%)	26 (52%)	
	Outside Providence	18 (42%)	2 (4%)	
During the past 9 months, how often did you	Think about leaving job (1 = never, 5 = always)	2.2 (1.2)	1.8 (0.9)	0.08
	Review other job opportunities (1 = never, 5 = always)	1.63 (0.93)	1.91 (1.02)	0.124
	Feel your values compromised (1 = never, 5 = always)	1.76 (0.75)	1.79 (0.8)	0.814
	Feel negative well-being (1 = never, 5 = always)	2.68 (1.08)	2.88 (1.15)	0.391
Job satisfaction	(0–100% rating)	73.4% (16.7)	80.8% (13.6)	**0.02**
Maslach scale measures	Emotional exhaustion (EE) total (0–54)	25.2 (12.3)	21 (10.5)	0.082
	Depersonalization (DEP) total (0–28)	11.6 (7.6)	8.8 (5.3)	**0.042**
	Personal achievement (PA) total (0–48)	37.3 (7.2)	37.3 (6.1)	0.963
	Burnout (from EE and DEP-0/1)	24 (55.8%)	16 (32%)	**0.024**
	Emotional exhaustion burnout (0/1)	20 (46.5%)	12 (24%)	**0.028**
	Depersonalization burnout (0/1)	20 (46.5%)	10 (20%)	**0.01**

The boldface values note statistical significance with a *p *< 0.05

**Fig. 2:**
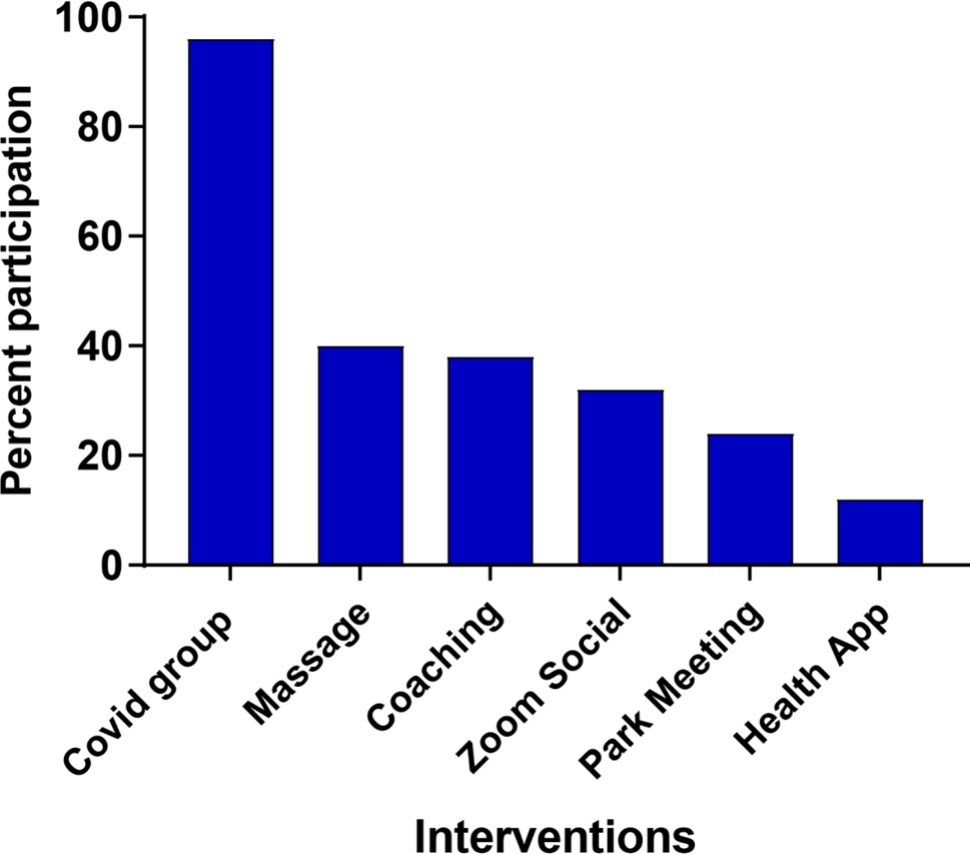
Percent participation in intervention activities by PPMC participants (from post-intervention survey)

Forty-eight percent of the intervention group reported high levels of wellness support vs. 0% of the control group (chi-square = 33.5, *p* < 0.00001). The intervention group attributed 44% of wellness support to Providence alone, while the controls attributed 12% to Providence alone (chi-square = 24.3, *p* < 0.00001). The interventions created high engagement. Ninety-six percent of survey respondents reported attending at least 1 of the COVID groups, and 72% reported attending 50% or more of the sessions. Thirty-eight percent of the hospitalists accessed 1:1 coaching. The least utilized intervention was the Behavioral Health appointments offered by the system at 12% (see Fig. 2). The impact was also noted when participants cited a variety of interventions as the most valuable at PPMC (see Table 4, participant quotes).

Statistical control of background characteristics did not reduce any observed intervention effects. Regression analyses of post-intervention survey data, in which sex, race, years of working experience, the presence of children under 18 in the home, and current working hours had forced equation entry, continued to show significant intervention effects for job satisfaction and burnout outcomes (Table 3). For all burnout outcomes, the intervention was the primary predictor, accounting for more variance (*R*^2^) than all other variables combined. In the equation predicting job satisfaction, the intervention was still the strongest predictor. However, post hoc inspection revealed a more complex pattern. If all medical providers with children under 18 were compared, control participants working part-time had lower job satisfaction (66%) than control parents working full-time (76%), intervention parents working part-time (80%), or intervention parents working full-time (81%).

**Table 3:** Regression Analyses

	Dependent outcomes			
	Job satisfaction	DEP burnout	EE burnout	Total* burnout
DF =	7, 84	7, 84	7, 84	7, 84
R2 (all terms)	0.19	0.23	0.12	0.13
R2 (PPMC/SVH)	.08	.15	.11	.08
Predictors	***p* value**	***p* value**	***p* value**	***p* value**
Sex (male/female)	0.28	0.85	0.55	0.99
Race (White vs other)	0.21	**0.027**	0.47	0.15
Child under 18 yr (Y/N)	**0.011**	0.78	0.19	0.29
Experience (6–15 yr)	0.067	0.35	0.64	0.11
Experience (> 15 yr)	0.28	0.72	0.59	0.39
Work hours	**0.018**	0.38	0.21	0.90
PPMC vs SVH^†^	**0.008**	**0.002**	**0.014**	**0.017**

The boldface values note statistical significance with a *p* < 0.05

*DEP* depersonalization, *EE* emotional exhaustion, *R2* amount of site-specific variance when included in the model with all other variables

^*^Sum of DEP and EE burnout

^†^Burnout intervention comparison

## Discussion

In this study, we demonstrated the effectiveness of implementing a multimodal behavioral intervention intended to mitigate physician burnout at an urban community hospital during the COVID pandemic. When participants were asked to identify our most important intervention, 67% indicated it was the Thursday meetings (see Table 4, participant quotes). If all meetings were attended, the cumulative duration of the Thursday meetings was 30 h over the year. Ninety-six percent of the intervention group participated in at least one meeting and noted multiple positive elements, including COVID treatment information, a running update on the number of admitted and discharged hospital patients, shared ways to increase efficiency, social support, and teambuilding. While we do not have the information necessary to precisely disentangle how the meetings helped hospital staff through specific struggles, an important element was the focus on controllable workplace elements and experience during the early pandemic months.

**Table 4:** Feedback from Participants—Most Valuable Parts of Intervention (from Post-intervention Survey, PPMC Only)

“The wellness group has done such a great job of identifying (really relevant) topics. I enjoy having a forum (thurs covid mtgs) to discuss things with other group members. The medical director group has been spectacular with providing regular updates and making sure that we are scheduled appropriately to avoid getting overworked during this stressful time.”
“Wellness meetings / Covid updates—even if only able to listen to the recording or read the recap—helps keep us on the same page!”
“Covid updates, gifts/massage—made me feel special and valuable at my work.”
“Although I did not have much coaching, I loved it. I love the COVID updates and discussions afterward. The care bag was sweet”
“I really enjoyed the zoom social for new hires, that helped me feel more accepted and included; I like the weekly covid/wellness meetings because they are helpful and offer great insight as well as good laughs”
“It’s a life line to know that we are supported during these times by the administration putting money into our wellness. It’s such a marker that they value our wellness especially while we’re taking care of Covid patients…I know as a group we feel supported and are stronger and healthier because of these interventions. Thank you so much.”

Anecdotally, one of the biggest shifts that we observed was increased clarity about the locus of control within the intervention group. In the beginning, we noted that most individuals and the group as a whole were unable to identify realistic and helpful interventions. We saw participant responses that conveyed learned helplessness or a sense of lack of control. COVID group development was accomplished through multiple study team meetings in which there was reflection on recent experiences followed by brainstorming about how to reduce stressors. This shifted the focus away from identifying uncontrollable variables to controllable ones. We had no control over how many COVID patients were in the hospital or availability of PPE, but we did have control over work flows and communication. We could share best practices among peers and challenges in caring for unvaccinated patients. This enabled individuals to set expectations that worked for them and were also embraced by the larger group.

Coaching was also cited as a novel and useful facet of the intervention (see Table 4, participant quotes). Thirty-eight percent of the members of the intervention group accessed 1:1 coaching. This one-on-one coaching was designed to empower individuals to create results they want within their personal sphere of control. This type of intervention and re-positioning of perspective has been proven as a helpful intervention to decrease burnout.^14–17,19^ Although we were unable to show superior outcomes in individuals who made use of available coaching, our inclusion of voluntary supportive coaching, when needed, as part of a multifaceted intervention adds to the literature on the utility of coaching in wellness programs.

Empowering hospitalists to identify and address their own stressors was associated with improved perceptions of organizational leadership even though our interventions were not designed for that purpose. We suspect that when individuals were empowered to support the group’s well-being, they were more likely to feel positively about their organization’s actions and to feel that leadership was honestly engaged in protecting their well-being. Hospitalists in the intervention group were much more likely to feel their wellness had been completely supported and that the support had come primarily from Providence rather than from outside the organization.

In addition to decreased burnout, job satisfaction was also significantly better at the intervention site. Although, at the end of the study period, there was not a statistically significant difference between the control group and the intervention group in the frequency with which they were considering leaving their job at the end, we speculate that the higher level of burnout and lower job satisfaction in the control may eventually lead to higher turnover and increased costs. While physician recruitment and replacement costs vary across specialties, workloads, and regions, the systemic consequences of losing even a single doctor are serious. Factoring in total costs (recruitment, interviews, relocation, lost revenue), multiple estimates have reported that it cost $500,000–1,000,000 to replace a single physician.^19–21^ Thus, decreasing physician burnout and improving job satisfaction is not simply a morale booster, but it is likely to be a major institutional cost savings, particularly during a pandemic. While our “thinking about leaving job” question was only borderline significant when comparing facilities, we observed that 5% of the control sample was “always” thinking about leaving, while none of the intervention group participants were.

The model of wellness warriors with paid, protected time has been critical for the intervention’s success. They were able to rapidly identify evolving stressors within their peer group and developed a growing expertise in addressing them. While there was a financial cost, it only equated to 0.28% of the total group FTE. Before this study, responsibility for the well-being of the group informally fell to the group leaders. However, these individuals have many competing demands for their time, and overall wellness was often deprioritized out of necessity as the pressures of the COVID pandemic stretched allocations thin. Through the use of Wellness Warriors and giving them protected time, we were able to support unit leaders. The Wellness Warriors were able to identify ongoing wellness needs of the PPMC hospitalist group, primarily through the weekly meetings, which in turn reduced the chronic demands on the hospitalist leadership. Thus, for a minor systemic cost, hospitalist burnout and leadership workloads were decreased and job satisfaction and confidence in leadership were increased. This adds to and strengthens the previously shared approach of empowering work units as a well-being strategy.^13,23^

This study had strengths and limitations. We controlled for many potentially confounding variables with regression analysis. Our results led us to believe that the intervention data are quite robust and indicative of the interventions’ impact rather than side effects of covariates or modifying variables, yet precise causes are difficult to determine. For confidentiality reasons, survey responses were anonymous, and comparisons of pre- and post-intervention responses can only be assessed at the facility level and we cannot say exactly which components of the meetings and other interventions were most useful for each individual. We also did not know exactly how long each participant was exposed to our interventions, although there is substantial overlap between the personnel present at these hospitals at the pre-and post-intervention surveys. Overall, our results indicate mean levels of job satisfaction and burnout being maintained among study participants in the intervention facility, while the control facility showed increased burnout and lower job satisfaction. Finally, there may be unmeasurable differences between the two sites that we were unable to adjust for in the multivariate analysis.

## Conclusion

Caregiver burnout, particularly during the COVID pandemic, continues to be a major challenge, and this study confirms that hospitalist burnout was exacerbated by the COVID-19 pandemic. Each physician and group experience unique challenges, and burnout drivers vary, which makes uniform system interventions difficult. However, this multifaceted approach demonstrated efficacy in reducing the prevalence of burnout and protecting job satisfaction. Since the end of the research period, COVID focused groups have continued to serve as an effective venue for addressing stressors while also building community. The group created during this research has continued, and meeting attendance and engagement remain high even 24 months after their creation. Creating a framework that embedded organizational change agents within a group resulted in increased feelings of organizational support, the perception that work hours were more manageable, and increased satisfaction with organizational leadership. The process of empowering and allocating resources and time for hospitalists to become Wellness Warriors created an infrastructure to address the continually changing needs in an unpredictable situation. This study adds to existing knowledge about the power of organizational support through protected time to address unique needs of a practicing medical group in addition to supporting individual employees with coaching. This model could be widely adopted across practice settings and specialties to address growing challenges of burnout in medical systems.

## Data Availability

The data that support the findings of this study are not openly available due to sensitive information which is subject to HIPPA restrictions. Additionally, the smaller N's in the groups examined could make identification of individuals possible. The data are available from the corresponding author upon reasonable request.

## Appendix

### COVID Groups

One of the primary interventions developed was the creation of COVID specific support groups. Our team realized that a major stressor for the providers was lack of information about up-to-date COVID treatments as well as work-flow changes. We created a virtual group meeting from 12–1 p.m. Thursdays on Microsoft TEAMS. The first 15 min of each meeting consisted of Infections Disease updates about disease patterns and treatments provided by the ID physician on service aligning with the groups shared value of providing up to date clinical care. The next 15 min was dedicated to work-flow updates and questions, in which Hospitalists on the COVID service would share helpful tips and tricks but also raise issues they were facing that were shared with leadership during the meeting. Initially, these meetings were held weekly. After 3 months, the meetings were held every other week. The first 15 min remained dedicated to ID updates and questions, but the remaining 45 min was tailored to the group’s immediate needs. During COVID surges, this varied between discussions about coping, debriefing difficult cases, updates from Intensive Care Unit physicians, and discussion of how to talk to patients hesitant about vaccines. During COVID nadirs an efficiency series was created where hospitalists that excelled in various aspects of Hospital medicine would share their strategies and work-flows. We also had sessions focused on community building where Hospitalists shared their passions outside of medicine or nurses were invited to share their experiences with the group. Each meeting topic was created and facilitated by the wellness team. Meeting minutes with pertinent highlights were emailed within 1 day, and most sessions were recorded for later viewing.

### Coaching

The primary investigator became certified through the Life Coach School, a thought-based coaching institution with individual and group training. Every interested hospitalist was offered 1:1 coaching from the primary investigator during the intervention period. There were unlimited free sessions available up to what was included in the 8 h of paid time per week supported through the grant.

#### Additional Support

As referenced above our system created behavioral health appointments where every employee could access visits with the behavioral health providers in our system for free. Each hospitalist also had access to the Employee Assistance Program.
